# The probabilistic no miracles argument

**DOI:** 10.1007/s13194-015-0122-0

**Published:** 2015-10-05

**Authors:** Jan Sprenger

**Affiliations:** Tilburg Center for Logic, Ethics and Philosophy of Science (TiLPS), Tilburg University, P.O. Box 90153, 5000 LE Tilburg, The Netherlands

**Keywords:** Scientific realism, No miracles argument, Bayesian epistemology, Base rate fallacy, Stability of scientific theories

## Abstract

This paper develops a probabilistic reconstruction of the No Miracles Argument (NMA) in the debate between scientific realists and anti-realists. The goal of the paper is to clarify and to sharpen the NMA by means of a probabilistic formalization. In particular, I demonstrate that the persuasive force of the NMA depends on the particular disciplinary context where it is applied, and the stability of theories in that discipline. Assessments and critiques of “the” NMA, without reference to a particular context, are misleading and should be relinquished. This result has repercussions for recent anti-realist arguments, such as the claim that the NMA commits the base rate fallacy (Howson (2000), Magnus and Callender (*Philosophy of Science*, *71*:320–338, 2004)). It also helps to explain the persistent disagreement between realists and anti-realists.

## Introduction

The debate between scientific realists and anti-realists is one of the classics of philosophy of science, comparable to a soccer match between Brazil and Argentina. Realism comes in different varieties, e.g., metaphysical, semantic and epistemological realism (see Chakravartty (2011), for a survey). In this paper, I focus on the epistemological thesis that we are justified to believe in the truth of our best scientific theories, and that they constitute knowledge of the external world (Boyd 1983; Psillos 1999, 2009). In this view, the existence of a mind-independent world (metaphysical realism) and the reference of theoretical terms to mind-independent entities (semantic realism) is usually presupposed—the real question concerns the epistemic status of our best scientific theories.

A major player in this debate is the No Miracles Argument (NMA). It contends that the truth of our best scientific theories is the only hypothesis that does not make the astonishing predictive, retrodictive and explanatory success of science a mystery (Putnam 1975, 73). If our best scientific theories did not correctly describe the world, why should we expect them to be successful? On the other hand, since the truth of our best theories is an excellent explanation of their success, we should accept the realist hypothesis: our best scientific theories are true and constitute knowledge of the world.

**Fig. 1:**
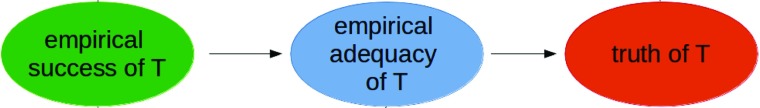
The structure of the NMA as a two-step argument from the empirical success of *T* to its truth. In our model, we conceptualize the NMA as an argument for the first inference in this figure, that is, for an inference from empirical success of *T* to its empirical adequacy

It is not entirely clear whether the NMA is an empirical or a super-empirical argument. As an argument from past and present success of our best scientific theories to their truth, it involves two major steps: the step from observed success to rational belief in empirical adequacy, and the step from rational belief in empirical adequacy to rational belief in truth (see Fig. 1). The first of them is an empirical inference, the second most probably not: ordinary empirical evidence cannot distinguish between different theoretical structures that yield the same observable consequences.

Much philosophical discussion has been devoted to the second step of the NMA (e.g., Psillos 1999; Lipton 2004; Stanford 2006), which seems in greater need of a philosophical defense. After all, the realist has to address the problems of underdetermination of theory by evidence and the existence of unconceived alternatives. But also the first step of the NMA is far from trivial, and strengthening it against criticism is vital for the scientific realist. For instance, Laudan (1981) has argued that there have been lots of successful, but non-referring scientific theories. If Laudan were right, then the entire NMA would break down, even if objections to the second step could be countered successfully.

Arguments against the first step of the NMA do not only threaten full scientific realists, but also structural realists (Worrall 1989) and some varieties of anti-realism that stick out their neck. One of them is Bas van Fraassen’s *constructive empiricism* (van Fraassen 1980; Monton and Mohler 2012). Proponents of this view deny that we have reasons to believe that our best scientific theories are literally true. However, they affirm that we are justified to believe in the *observable* parts of our best theories. Thus they are also affected by criticism and defense of the first step of the NMA.

Hence, the first step of the NMA does not draw a sharp divide between realists and anti-realists. Rather, the debate takes place between those who derive epistemic commitments from the success of science, and those who deny them. For convenience, we stick to the traditional terminology and refer to the first group as “realists” and to the second group as “anti-realists”.

The paper aims to show the realist and anti-realist standpoints can be reconciled with probabilistic rationality, and Bayesian rationality in particular. The paper assumes that rationally believed propositions should also be probable (for discussion of this thesis, see e.g., Leitgeb 2014). I set up a simple probabilistic model of the NMA and use it to underline a recent criticism by Colin Howson (2000) and Howson (2013) that the NMA falls prey to the base rate fallacy (Section 2). I then develop a refined probabilistic model of the NMA where this criticism is addressed, and where the stability of scientific theories and the existence of relevant alternatives are accommodated (Section 3). By assigning a crucial role to the nature and history of the relevant scientific discipline, this model allows for a more nuanced assessment of the NMA. The paper concludes with a short discussion of the scope of the NMA, and a plea for context-sensitive assessment of its validity (Section 4).

## A probabilistic no miracles argument

**Fig. 2:**
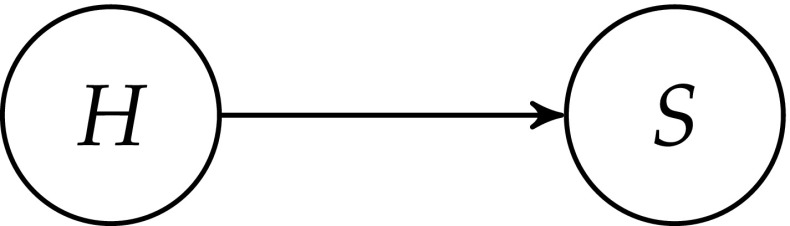
The Bayesian Network representation of the impact of *H*—the empirical adequacy of theory *T*—on the empirical success of *T*, denoted by *S*

The NMA is sometimes understood as a *general argument* for believing in the truth of our best scientific theories. In this paper, we restrict it to a *particular scientific theoryT* which is predictively and explanatorily successful in a certain scientific domain. Since we only investigate arguments for the *empirical adequacy* of *T*, we introduce a propositional variable *H*—the hypothesis that *T* is empirically adequate. See Fig. 2 for a simple Bayesian network representation of the dependence between *H* and the propositional variable *S* that represents the empirical success of *T*.

Expressed as a probabilistic argument, the simple NMA then runs as follows: *S* is much more probable if *T* is empirically adequate than if it is not:

$$p(S|H) \gg p(S|\neg H) $$

From Bayes’ Theorem, we can then infer

$$p(H|S) > p(H) $$

In other words, *SconfirmsH*: our degree of belief in the empirical adequacy of *T* is increased if *T* is successful.^1^ Here, we have interpreted the above probabilities as rational degrees of beliefs, and the NMA as proving an increase in degree of belief. This subjective Bayesian interpretation of the NMA strikes us as natural. However, the validity of the argument is not affected if an objective interpretation of probability is adopted instead. This is the reason why I refer to the *probabilistic* rather than the Bayesian NMA. I leave the preferred interpretation of probability to the reader’s taste and do not consider this issue further.

To the above argument, anti-realists object that the inequality *p*(*H*|*S*)>*p*(*H*) falls short of establishing the realist claim. We are primarily interested in whether *H* is *sufficiently probable givenS*, not in whether *S* raises the probability of *H*. After all, the increase in probability could be negligibly small. The result *p*(*H*|*S*)>*p*(*H*) does not establish that *p*(*H*|*S*) is beyond a critical threshold, e.g., a probability of 1/2.

More specifically, it has been argued that the NMA commits the *base rate fallacy* (Howson 2000; Magnus and Callender 2004). This is an unwarranted type of inference that frequently occurs in medicine. Consider a highly sensitive medical test which yields a positive result. On the other hand, the medical condition in question is very rare, that is, the base rate of the disease is very low. In such a case, the posterior probability of the patient having the disease, given the test, will still be quite low. Nonetheless, medical practitioners tend to disregard the low base rate and to infer that the patient really has the disease in question (e.g., Goodman 1999).

This objection can be elucidated by a brief inspection of Bayes’ Theorem. Our quantity of interest is the posterior probability *p*(*H*|*S*), our confidence in *H* given *S*. This quantity can be written as

1 $$\begin{array}{@{}rcl@{}} p(H|S) &=& \frac{p(H) \; p(S|H)}{p(S)} \\ &=& \left( 1 + \frac{1 - p(H)}{p(H)} \frac{p(S|\neg H)}{p(S|H)} \right)^{-1} \end{array} $$

which shows that *p*(*H*|*S*) is not only an increasing function in *p*(*S*|*H*) and a decreasing function in *p*(*S*|¬*H*): its value crucially depends on the base rate or prior plausibility of *H*, *p*(*H*) (=the probability that *T* is empirically adequate).

Anti-realists claim that NMA is built on a base rate fallacy: from the high value of *p*(*S*|*H*) (“the empirical adequacy of *T* explains its success”) and the low value of *p*(*S*|¬*H*) (“success of *T* would be a miracle if *T* were not empirically adequate”), rational belief in *H* (“*T* is empirically adequate”) is inferred. The probabilistic model of the NMA demonstrates that we need additional assumptions about *p*(*H*) to warrant this inference. In the absence of such assumptions, the NMA does not entitle us to accept *T* as empirically adequate.

What do these considerations show? First, they expose that the NMA, reconstructed as a probabilistic inference to the posterior probability of *H*, is essentially subjective. After all, any weight of evidence in favor of *H* can be counterbalanced by a sufficiently skeptical prior, that is, a sufficiently low value assigned to *p*(*H*). The anti-realist contends that the realist needs to provide convincing reasons why *p*(*H*) should not be arbitrarily close to zero, and that such reasons will typically presuppose realist inclinations. This is a problem for those realists who claim that the NMA is an *objective* argument in favor of scientific realism. Howson (2013, 211) concludes that due to the dependence on unconstrained prior degrees of belief, the NMA is, “as a supposedly objective argument, […] dead in the water”. See also Howson (2000, ch. 3), Lipton (2004, 196–198), and Chakravartty (2011).

Second, the anti-realist may argue that empirical adequacy is not required for predictive success. Every now and then, science undergoes radical discontinuities: it is discovered that central terms in scientific theories do not refer, that theories fail to apply outside a particular domain, etc. The theories which supersede them are empirically distinct. Why should our currently best theory *T*_*n*_=*T* not suffer the same fate as it predecessors *T*_1_,…,*T*_*n*−1_ which proved to be empirically inadequate although they once were the best scientific theory? This is basically Laudan’s argument from Pessimistic Meta-Induction (PMI): “I believe that for every highly successful theory in the past of science which we now believe to be a genuinely referring theory, one could find half a dozen successful theories which we now regard as substantially non-referring” (Laudan 1981, 35). See (Tulodziecki, unpublished) for a recent case study.

PMI affects the values of *p*(*S*|¬*H*) and *p*(*H*) as follows: On the one hand, history teaches us that there have often been false theories that explained the data well (and were superseded later). In other words, empirically inadequate theories can be highly successful and *p*(*S*|¬*H*) need not be that low. Second, the fact that *T*_1_,…,*T*_*n*−1_ stand refuted, plus possible continuities and structural similarities between those theories and *T*_*n*_=*T*, suggest, by virtue of an inductive inference, that *T* may ultimately suffer the same fate, lowering the probability that *T* is empirically adequate.

Let us check these arguments in a numerical analysis of the probabilistic NMA. Conceding a bit to the realist, we set *s*:=*p*(*S*|*H*)=1: if theory *T* is empirically adequate, then it is also successful.^2^ Furthermore, define *s*^′^:=*p*(*S*|¬*H*) and let *h*:=*p*(*H*) be the prior probability of *H*. We now ask the question: for which values of *s*^′^ and *h* is the posterior probability of *H*, *p*(*H*|*S*), greater than 1/2? That is, when would it be more plausible to believe that *T* is empirically adequate than to deny it? This condition is arguably a minimal requirement for the claim that the success of *T* entitles us to rational belief in its empirical adequacy.

By using Bayes’ Theorem, we can easily calculate when the inequality *p*(*H*|*S*)>1/2 is satisfied. Equation 1 brings us to the inequality

$$\frac{1}{2} < \left( 1 + s^{\prime} \frac{1 - h}{h} \right)^{-1} $$

which can be written as

2 $$ s^{\prime} < \frac{h}{1-h} $$

**Fig. 3:**
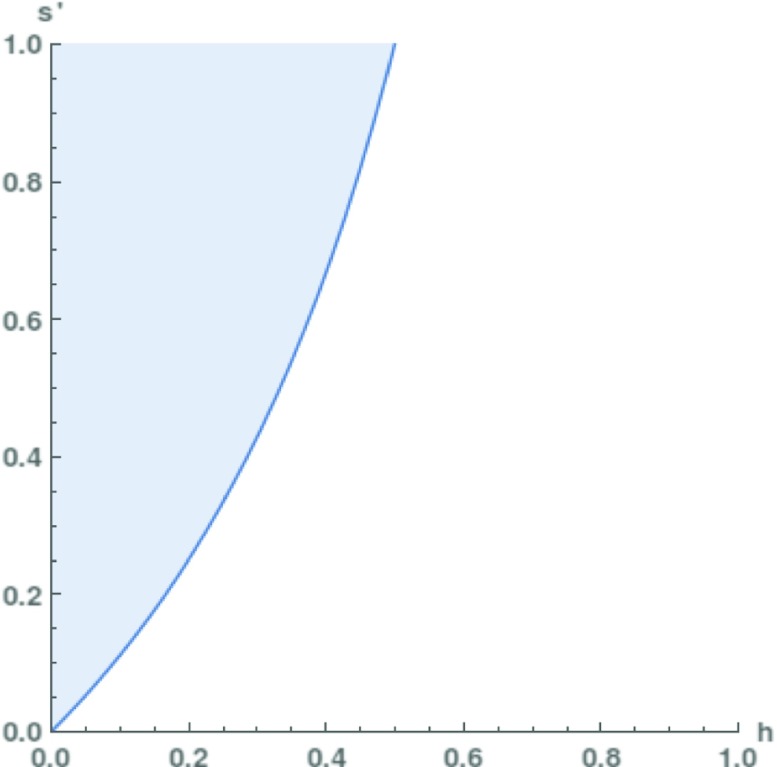
The scope of the No Miracles Argument, represented graphically. *p*(*H*|*S*)>1/2 is the case in the white area below the line

See Fig. 3 for a graphical illustration.

However, inequality (2) is not easy to satisfy. As mentioned above, false theories and models often make accurate predictions and perform well on other cognitive values (see Frigg and Hartmann 2012, for an overview). Classical examples that are still used today involve Newtonian mechanics, the Lotka-Volterra model from population biology (e.g., Weisberg 2007) and Rational Choice Theory. These theories may be false, but they are definitely useful and successful. Given the frequency of such theories in science, we may estimate *s*^′^=1/4—the precise value matters less than the order of magnitude. To satsify inequality (2) and to make the NMA work, we would then require that *p*(*H*)∈[1/3,1]! In other words, the NMA only flies for theories with a substantial prior probability of being empirically adequate. What is more, for a “mildly skeptical prior” such as *p*(*H*)=0.05, the value of *s*^′^ would have to be in the range [0,0.05]. This amounts to making the assumption that only the empirical adequacy (or truth) of a scientific theory can explain its success. But this is essentially a realist premise which the anti-realist would refuse to accept. She could point to the existence of unconceived alternatives (Stanford 2006, ch. 6), the explanatory successes of false theories, etc. In other words: the simple probabilistic model of the NMA demonstrates that (1) to the extent that the NMA is valid, its premises presuppose realist inclinations; (2) to the extent that the NMA builds on premises that are neutral between the realist and the anti-realist, it fails to be valid.

Are things thus hopeless for the realist who wants to convince the anti-realist that the NMA is a good argument? Does “all realistic hope of resuscitating the [no miracles] argument [fail]”, as Howson (2013, 211) writes? Perhaps not necessarily so. The next section develops another, more sophisticated probabilistic model whose results are more friendly toward the realist position.

## A new model of the no miracles argument

So far, the probabilistic NMA only took the predictive and explanatory success of *T* as evidence for the realist position. But perhaps, different kinds of evidence bear on the argument, too. For example, Ludwig Fahrbach (2009, 2011) has argued that the stability of scientific theories in recent decades favors scientific realism. In this section, we show how such arguments could be part of a probabilistic NMA. We do not want to take a stand on the historical correctness of Fahrbach’s observations: rather, we would like to demonstrate how such observations can *in principle* affect the NMA.

Fahrbach’s argument is based on scientometric data. He observes an exponential growth of scientific activity, with a doubling of scientific output every 20 years (Meadows 1974). He also notes that at least 80 % of all scientific work has been done since the year 1950 and observes that our fundamental scientific theories (e.g., the periodic table of elements, optical and acoustic theories, the theory of evolution, etc.) were stable during that period of time. That is, they did not undergo rejection or major conceptual change. Laudan’s examples in favor of PMI, on the other hand, all stem from the early periods of science: the caloric theory of heat, the ether theory in physics, or the humoral theory in medicine.

For giving a fair assessment of PMI, we have to take into account the amount of scientific work done in a particular period. This implies, for example, that the period 1800–1820 should receive much less weight than the period 1950–1970. According to Fahrbach, PMI fails because most “theory changes occurred during the time of the first 5 % of all scientific work ever done by scientists” (Fahrbach 2011, 149). If PMI were valid, we should have observed more substantial theory changes or scientific revolutions in the recent past. However, although the theories of modern science often encounter difficulties, revolutionary turnovers do not (or only very rarely) happen. According to Fahrbach, PMI stands refuted—or at the very least, it is not more rational than *optimistic* meta-induction.

The factual correctness of Fahrbach’s observations may be disputed, and his model is certainly very simplified. Yet, it deserves to be taken seriously, particularly with respect to the implications for the NMA. In this paper, I explore if observations of long-term stability expand the set of circumstances where the NMA holds. To this end, let us refine our probabilistic model of the NMA.

As before, the propositional variable *H* expresses the empirical adequacy of theory *T*, and *S* denotes the predictive, retrodictive and explanatory success of *T*. Similar to Dawid et al. (2015), we introduce a integer-valued random variable *A* that expresses the number of satisfactory alternatives to *T*, including unconceived theories. In the individuation of alternatives, we stick with the Dawid et al. paper: that is, we demand that alternative theories satisfy a set of (context-dependent) theoretical constraints $\mathcal {C}$, are consistent with the currently available data $\mathcal {D}$, and give distinguishable predictions for the outcome of some set $\mathcal {E}$ of future experiments. In line with our focus on empirical adequacy rather than truth, we do not distinguish between empirically equivalent theories with different theoretical structures. Finally, major theory change in the domain of *T* is denoted by variable *C*, and absence of change and theoretical stability by ¬*C*. “Theory change” is understood in a broad sense, including scenarios where rivalling theories emerge and end up co-existing with *T*.

**Fig. 4:**
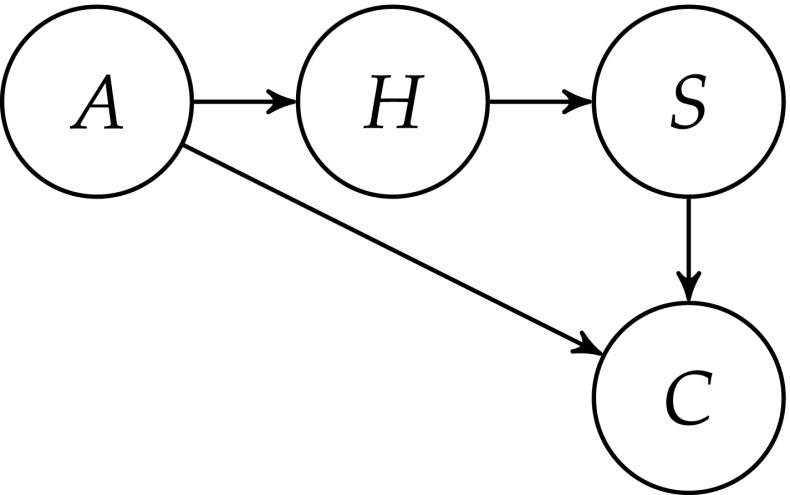
The Bayesian Network representation of the relation between variables *A* (the number of alternatives to *T*), *H* (empirical adequacy of theory *T*), *S* (the success of *T*) and *C* (major theory change)

The dependency between these four propositional variables—*A*, *C*, *H* and *S*—is given by the Bayesian network in Fig. 4. *S*, the success of theory *T*, only depends on the empirical adequacy of *T*, that is, on *H*. The probability of *H* depends on the number of distinct alternatives that are also consistent with the current data, etc. Finally, *C*, the probability of observing substantial theory change, depends on *S* and *A*: the empirical success of *T* and the number of available alternatives to *T* that scientists might develop. *H* affects *C* only via *A*. All this is captured in the following assumption:

A0 The variables *A*, *C*, *H* and *S* satisfy the conditional independencies in the Bayesian Network of Fig. 4.

To rule out preservation of a theory by a of series degenerative accommodating moves, the variable *C* should be evaluated over a longer period (e.g., 30–50 years). We now define a number of real-valued variables in order to facilitate calculations:

- Denote by *a*_*j*_:=*p*(*A*=*j*) the probability that there are exactly *j* (not necessarily discovered) alternatives to *T* that satisfy the theoretical constraints $\mathcal {C}$, are consistent with current data $\mathcal {D}$ and give definite predictions for future experiments $\mathcal {E}$, etc.^3^
- Denote by *h*_*j*_:=*p*(*H*|*A*=*j*) the probability that *T* is empirically adequate if there are exactly *j* alternatives to *T*.
- As before, denote by *s*:=*p*(*S*|*H*) and *s*^′^:=*p*(*S*|¬*H*) the probability that *T* is successful if it is (not) empirically adequate.
- Denote by *c*_*j*_:=*p*(¬*C*|*A*=*j*,*S*) the probability that no substantial theory change occurs if *T* is successful and there are exactly *j* alternatives for *T*.

Suppose that we now observe ¬*C* (no substantial theory change has occurred in the last decades) and *S* (theory *T* is successful). The Bayesian network structure allows for a simple calculation of the posterior probability of *H*:

$$\begin{array}{@{}rcl@{}} p(\neg CSH) &=& \sum\limits_{A} p(A) p(\neg C|AS) p(S|H) p(H|A) \\ &=& \sum\limits_{j=0}^{\infty} a_{j} \, c_{j} \, s \, h_{j} \\ p(\neg CS) &=& {\sum}_{A, H} p(A) p(\neg C|AS) p(S|H) p(H|A) \\ &=& \sum\limits_{A} p(A) p(\neg C|AS) p(S|H) p(H|A) + \sum\limits_{A} p(A) p(\neg C|AS) p(S|\neg H) p(\neg H|A) \\ &=& \sum\limits_{j=0}^{\infty} a_{j} \, c_{j} \, (s \, h_{j} + s^{\prime} (1-h_{j})) \end{array} $$

With the help of Bayes’ Theorem, these equations allows us to calculate the posterior probability of *H* conditional on *C* and *S*. That is, how probable is *H* given that *T* is successful and that we have observed no major theoretical change in recent decades?

3 $$ p(H|\neg CS) = \frac{p(\neg CSH)}{p(\neg CS)} = \frac{\sum\nolimits_{j=0}^{\infty} a_{j} \, c_{j} \, s \, h_{j}}{\sum\nolimits_{j=0}^{\infty} a_{j} \, c_{j} \, (s \, h_{j} + s^{\prime} (1-h_{j}))} $$

We now make some assumptions on the values of these quantities.

1. If *T* is empirically adequate then it will be successful in the long run: *s*=*p*(*S*|*H*)=1.^4^
2. The empirical adequacy of *T* is no more or less probable than the empirical adequacy of an alternative which satisfies the same set of theoretical and empirical constraints: *h*_*j*_:=*p*(*H*|*A*_*j*_)=1/(*j*+1). Scientists are indifferent between theories which satisfy the same set of theoretical and empirical constraints.
3. The more satisfactory alternatives are in principle accessible to the scientists, the less likely is an extended period of theoretical stability. In other words, *c*_*j*_:=*p*(¬*C*|*A*=*j*,*S*) is a decreasing function of *j*. For convenience, we choose *c*_*j*_=1/(*j* + 1).

While A2 is a default assumption made for expositional ease, A3 is more substantive: ceteris paribus, the probability of long-term theoretical stability decreases the more satisfactory alternatives for a successful theory exist. This qualitative claim is very intuitive. More contentious is the precise rate of decline. That’s why we will relax the peculiar assignment of the *c*_*j*_ later on in the paper.

A4 Assume that *T* is our currently best theory and we happen to find a satisfactory alternative *T*^′^. Then, the probability of finding yet another alternative *T*^″^ is the same as the probability of finding *T*^′^ in the first place. Formally:
  4 $$ p(A>j|A \ge j) = p(A>j+1|A \ge j+1) \; \forall j \ge 0. $$
  In other words, Eq. 4 expresses the idea that the number of known alternatives does not, in itself, raise or lower the probability of finding another alternative.

Note that A0–A4 are equally plausible for the realist and the anti-realist. In other words, no realist bias has been incorporated into the assumptions. We can now show the following proposition (proof in the Appendix):

### **Proposition 1**

*If a*_0_*>0, then A4 is equivalent to the requirement a*_*j*_*:=a*_0_*⋅(1−a*_0_*)*^*j*^.

Together with this proposition, A0–A4 allow us to rewrite Eq. 3 as follows:

5 $$ p(H|\neg CS) = \frac{\sum\nolimits_{j=0}^{\infty} (1-a_{0})^{j} \, \frac{1}{(j+1)^{2}}} {\sum\nolimits_{j=0}^{\infty} (1-a_{0})^{j} \, \frac{1-s^{\prime}j}{(j+1)^{2}}} $$

**Fig. 5:**
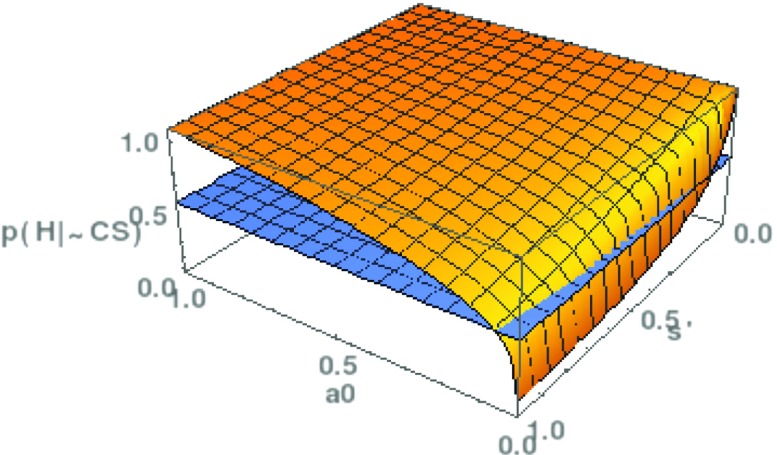
The scope of the No Miracles Argument in the revised formulation. The posterior probability of *H*, *p*(*H*|¬*C* *S*), is plotted as a function of (1) the prior probability that there are no satisfactory alternatives to *T* (*a*_0_); (2) the probability that *T* is successful if *T* is false ($s^{\prime } = p(S|\neg H)$). The hyperplane *z*=1/2 is inserted in order to show for which parameter values *p*(*H*|¬*C* *S*) is greater than 1/2

With the help of this formula, we can now rehearse the NMA once more and determine its scope, that is, those parameter values where *p*(*H*|¬*CS*)>1/2. The two relevant parameters are *a*_0_, the probability that there are no satisfactory alternatives to *T*, and *s*^′^, the probability that *T* is successful if it is not empirically adequate. Since an analytical solution of Eq. 5 is not feasible, we conduct a numerical analysis. Results are plotted in Fig. 5.

These results are very different from the ones in Section 2. With the hyperplane *z*=0.5 dividing the graph into a region where *T* may be accepted and a region where this is not the case, we see that the scope of the NMA has increased substantially compared to Fig. 3. For instance, *a*_0_>0.1 suffices for a posterior probability greater than 1/2, even if the value of *s*^′^ is very high. This is a striking difference to the previous analysis where way more optimistic values had to be assumed in order to make the NMA work.

So far, the analysis has been conducted in terms of *absolute confirmation*, that is, the posterior probability of *H*. We now complement it by an analysis in terms of *relative* or *incremental confirmation*. That is, we calculate the degree of confirmation that ¬*CS* exerts on *H*. We use four different measures to show the robustness of our results. First, we calculate the log-likelihood measure *l*(*H*,*E*)= log2*p*(*E*|*H*)/*p*(*E*|¬*H*) which has a good reputation in formal theories of evidence (e.g., Hacking 1965; Fitelson 2001) and a firm standing in scientific practice (e.g., Royall 1997; Good 2009). To apply it to the present case, we calculate

$$\begin{array}{@{}rcl@{}} p(\neg CS|H) = \frac{p(\neg CSH)}{p(H)} &=& \frac{{\sum}_{A} p(A) p(\neg C|AS) p(S|H) p(H|A)}{{\sum}_{A} p(A) \, p(H|A)} \\ &=& \frac{\sum\nolimits_{j=0}^{\infty} a_{j} \, c_{j} \, s \, h_{j}}{\sum\nolimits_{j=0}^{\infty} a_{j} \, h_{j}} \\ &=& \frac{{\sum}_{j=0}^{\infty} (1-a_{0})^{j} \frac{1}{(1+j)^{2}}}{\sum\nolimits_{j=0}^{\infty} (1-a_{0})^{j} \frac{1}{1+j}} \end{array} $$

$$\begin{array}{@{}rcl@{}} p(\neg CS|\neg H) = \frac{p(\neg CS \neg H)}{p(\neg H)} &=& \frac{\sum\nolimits_{A} p(A) p(\neg C|AS) p(S|\neg H) p(\neg H|A)}{\sum\nolimits_{A} p(A) \, p(\neg H|A)} \\ &=& \frac{\sum\nolimits_{j=0}^{\infty} a_{j} \, c_{j} \, s^{\prime} \, (1-h_{j})}{\sum\nolimits_{j=0}^{\infty} a_{j} \, (1-h_{j})} \\ &=& \frac{\sum\nolimits_{j=0}^{\infty} (1-a_{0})^{j} \frac{s^{\prime}j}{(1+j)^{2}}}{\sum\nolimits_{j=0}^{\infty} (1-a_{0})^{j} \frac{j}{1+j}} \end{array} $$

*l*(*H*,*E*) is a confirmation measure that describes the *discriminative power* of the evidence with respect to the realist and the anti-realist hypothesis. It is relatively insensitive to prior probabilities. Other measures aim at quantifying the increase in degree of belief from *p*(*H*) to *p*(*H*|*E*). Typical representatives of that class are the log-ratio measure *r*(*H*,*E*), the difference measure *d*(*H*,*E*) and the Crupi-Tentori measure *z*(*H*,*E*). The definitions of all measures are given below (see also Crupi 2013), and their values follow straightforwardly from the above calculations.

$$\begin{array}{@{}rcl@{}} l(H, E) &=& \log_{2} \frac{p(E|H)}{p(E|\neg H)} \quad\,\,\,\,{\kern.5pt} r(H, E) = \log_{2} \frac{p(H|E)}{p(H)} \\ d(H, E) &=& p(H|E) - p(H) \quad z(H, E) = \frac{p(H|E) - p(H)}{1-p(H)} \;\; \text{(if \(p(H|E) > p(H)\))} \end{array} $$

To deliver a comprehensive picture and to show the robustness of our claims, we calculate the degree of confirmation for all four confirmation measures. All measures are normalized such that a value of zero corresponds to neither confirmation nor disconfirmation. In addition, *d*(*H*,*E*) and *z*(*H*,*E*) only take values between -1 and 1.

**Fig. 6:**
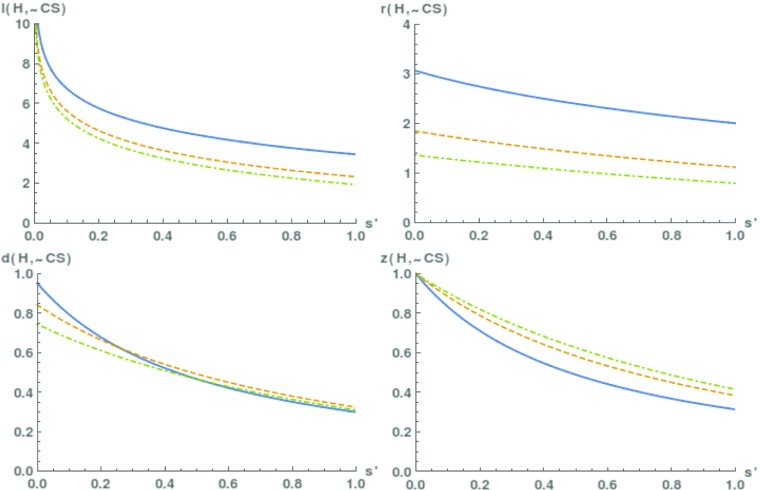
The degree of confirmation *l*(*H*,¬*C* *S*), *r*(*H*,¬*C* *S*), *d*(*H*,¬*C* *S*) and *z*(*H*,¬*C* *S*), for three different values of *a*_0_. Full line: *a*_0_=0.01. Dashed line: *a*_0_=0.05. Dot-dashed line: *a*_0_=0.1

Figure 6 plots the degree of confirmation as a function of the value of *s*^′^, for three different values of *a*_0_, namely 0.01, 0.05 and 0.1. As visible from the graph, the degree of confirmation is substantial for all four measures. In particular, it is robust vis-à-vis the values of *a*_0_ and *s*^′^, contrary to the anti-realist argument from Section 2. For example, if *s*^′^ is quite small, as it will often be the case in practice, then *l*(*H*,¬*CS*) comes close to 10, which corresponds to a ratio of more than 1,000 between *p*(¬*CS*|*H*) and *p*(¬*CS*|¬*H*)! This finding accounts for the realist intuition that the stability of scientific theories over time, together with their empirical success, is strong evidence for their empirical adequacy.

Finally, we conduct a robustness analysis regarding A3. Arguably, the function *c*_*j*_:=*p*(¬*C*|*A*=*j*,*S*)=1/(*j*+1) suggests that scientists are quite ready to give up on their currently best theory in favor of a good alternative. But as many have philosophers and historians of science have argued (e.g., Kuhn 1977), scientists may be more conservative and prefer the traditional framework, even if good alternatives exist. Therefore we also analyze a different choice of the *c*_*j*_, namely $c_{j} := e^{-\frac {1}{2} \left (\frac {j}{\alpha } \right )^{2}}$, where *c*_*j*_ falls more gently in *j*. This leads to the following expression for the posterior probability of *H*:

$$p(H|\neg CS) = \frac{{\sum}_{j=0}^{\infty} (1-a_{0})^{j} \, \frac{1}{j+1} e^{-\frac{1}{2} \left( \frac{j}{\alpha} \right)^{2}}} {{\sum}_{j=0}^{\infty} (1-a_{0})^{j} \, \frac{1-s^{\prime}j}{j+1} \, e^{-\frac{1}{2} \left( \frac{j}{\alpha} \right)^{2}}} $$

**Fig. 7:**
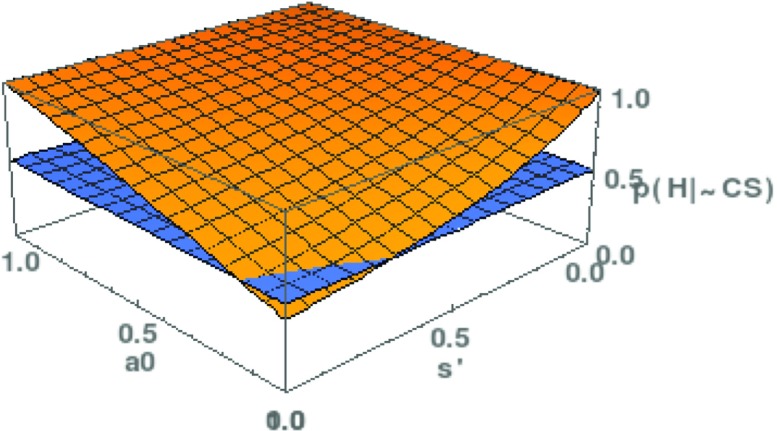
The scope of the No Miracles Argument in the revised formulation, with $c_{j} := e^{-\frac {1}{2} \left (\frac {x}{\alpha } \right )^{2}}$. The posterior probability of *H*, *p*(*H*|¬*C* *S*), is plotted as a function of *a*_0_ and $s^{\prime }$, like in Fig. 5, and contrasted with the hyperplane *z*=1/2

The graph of *p*(*H*|¬*CS*), as a function of *a*_0_ and *s*^′^, is presented in Fig. 7. We have set *α*=4, which corresponds to a gradual decline of *c*_*j*_. Yet, the results match those from Fig. 5: the scope of the NMA is much larger than in the simple version of the probabilistic NMA. Hence, our findings seem to be robust toward different choices of *c*_*j*_. To achieve a result similar to the one for the primitive NMA (Fig. 3), one would have to choose *α*=8, which will only be the case if scientists are really reluctant to reject the currently best theory. In such circumstances, stability is the default state of a discipline and will not strongly support the NMA.

All in all, our model shows that a probabilistic NMA need not be doomed. Its validity depends crucially on the properties of the disciplinary context where it operates in. This involves the existence of satisfactory alternatives and whether or not the discipline has been in a long period of theoretical stability. Of course, our model makes simplifying assumptions, but unlike those in Section 2, they do not carry a realist bias. This allows for a more nuanced and context-sensitive assessment of realist claims.

## Conclusions

This paper has investigated scope and limits of the No Miracles Argument (NMA) when formalized as a probabilistic argument. In the simple probabilistic model of the NMA, we have confirmed Howson’s (2000, 2013) verdict that it does not hold water as an objective argument: too much depends on the choice of the prior probability *p*(*H*), assuming what is supposed to be shown. We have supported this diagnosis by a detailed analysis of the probabilistic mechanics of NMA.

However, not all is lost for the NMA. We have developed a probabilistic model of the NMA where additional evidence, such as the stability of scientific theories, can be accommodated. This model also conceptualizes the possible alternatives to the theory in question. Using *this* model, scientific realism can be defended with much weaker assumptions than in the simple probabilistic version of the NMA. While context-sensitive assumptions are required in this version of the NMA, their relative weakness leaves open the possibility of a coherent, non-circular realist position in philosophy of science. See also (Dawid and Hartmann, unpublished).

I would like to stress that the context-sensitive nature of the NMA is not a vice, but a virtue. True, context-sensitive elements undermine the persuasive force of the NMA for those who do not already share realist inclinations. But when there is little stability in our currently best theories, or when empirically successful alternatives abound, there is nothing to feed realist intuitions in the first place. Hence it is natural that contextual considerations determine when the NMA is valid and when it isn’t.

Our analysis in Section 3 has shown how much depends on the stability of scientific theories in order to make the NMA fly. More research is needed into which areas of science have been theoretically stable. It may be argued that scientific theories of the recent past remained stable on the surface, e.g., in the basic assumptions they make, but that central concepts tacitly underwent major meaning shifts. Evolutionary biology is such a case in question: while basic principles such as the causal power of natural selection to bring about evolutionary change have been unchanged, the meaning of concepts such as natural selection, fitness, and selective environments has been fiercely debated (Lloyd 2014).

All this demonstrates that conceptualizations of the NMA as a *general* argument for scientific realism are mistaken. Instead of reading NMA as a “wholesale argument” for scientific realism that is valid across the board, we should understand it as a “retail argument” (Magnus and Callender 2004), that is, as an argument that may be strong for some scientific theories and weak for others.

For philosophers like myself, who are not committed to a particular position in the debate between realists and anti-realists, the probabilistic reconstruction of the NMA offers the chance to understand the argumentative mechanics behind the realist intuition, to better appreciate the context-dependency of the NMA, and to critically evaluate the merits of realist and anti-realist standpoints. The realist argument is based on contexts where theories are stable and there are few potential explanations of empirical success. Such circumstances favor the NMA. The anti-realist objections are grounded on those case studies where scientific theories have been volatile or one of our assumptions A0–A4 is implausible. The probabilistic reconstruction of the NMA can thus explain and guide the strategies that realists and anti-realists pursue when defending their positions.

## Appendix:: Proof of Proposition 1

We begin with the “ ⇒” direction of the proposion. Assumption A4 is equivalent to the following claim:

$$p(A>j|\bigvee_{k=j}^{\infty} (A=k)) = p(A>j+1|\bigvee_{k=j+1}^{\infty} (A=k)) \; \forall j \ge 0 $$

which is again equivalent to

$$p(A=j|\bigvee_{k=j}^{\infty} (A=k)) = p(A=j+1|\bigvee_{k=j+1}^{\infty} (A=k)) \; \forall j \ge 0 $$

This entails that for all *j*≥0, we have

$$\frac{p(A=j)}{p(\bigvee_{k=j}^{\infty} (A=k))} = \frac{p(A=j+1)}{p(\bigvee_{k=j+1}^{\infty} (A=k))} $$

This implies in turn

$$\begin{array}{@{}rcl@{}} p(A=j+1) &=& p(A=j) \, \frac{p(\bigvee_{k=j+1}^{\infty} (A=k))}{p(\bigvee_{k=j}^{\infty} (A=k))} \\ &=& p(A=j) \, \frac{ 1-{\sum}_{k=0}^{j} p(A=k) } {1-{\sum}_{k=0}^{j-1} p(A=k)} \end{array} $$

By a simple induction proof, we can now show

6 $$ p(A=n) = p(A=0) \, \left( 1- {\sum}_{k=0}^{n-1} p(A=k) \right) $$

For *n*=1, Eq. 6 follows immediately. Assuming that it holds for level *n*, we then obtain

$$\begin{array}{@{}rcl@{}} p(A=n+1) &=& p(A=n) \, \frac{1-\sum\nolimits_{k=0}^{n} p(A=k) } { 1-\sum\nolimits_{k=0}^{n-1} p(A=k) } \\ &=& p(A=0) \, \left( 1- \sum\limits_{k=0}^{n-1} p(A=k) \right) \, \frac{1-\sum\nolimits_{k=0}^{n} p(A=k) } {1-\sum\nolimits_{k=0}^{n-1} p(A=k)}\\ &=& p(A=0) \left( 1-\sum\limits_{k=0}^{n} p(A=k) \right) \end{array} $$

where we have used the inductive premise in the second step. Finally, we use straight induction once more to show that

7 $$ p(A=n) = p(A=0) (1-p(A=0))^{n} $$

where the case *n*=0 is trivial and the inductive step *n*→*n*+1 is proven as follows:

$$\begin{array}{@{}rcl@{}} p(A=n+1) &=& p(A=0) \, \left( 1-\sum\limits_{k=0}^{n} p(A=k) \right) \\ &=& p(A=0) \, \left( 1- \sum\limits_{k=0}^{n} p(A=0) \, (1-p(A=0))^{k} \right) \\ &=& p(A=0) \left( 1- p(A=0) \frac{1-(1-p(A=0))^{n+1}}{1-(1-p(A=0))} \right) \\ &=& p(A=0) (1- (1 - (1-p(A=0))^{n+1})) \\ &=& p(A=0) (1-p(A=0))^{n+1} \end{array} $$

In the second line, we have applied the inductive premise to *p*(*A*=*k*), and in the third line, we have used the well-known formula for the geometric series:

8 $$ \sum\limits_{k=0}^{n} q^{k} = \frac{1-q^{n+1}}{1-q} $$

This shows Eq. 7 and completes the proof of the “ ⇒” part of the proposition. The ” ⇐” direction is a purely algebraic exercise: using Eq. 8 and the fact that $\sum \nolimits _{k=0}^{\infty } a_{k} =1$, we can easily calculate that

$$p(A \ge j) = (1-a_{0})^{j} \qquad\quad p(A \ge j+1) = (1-a_{0})^{j+1} $$

from which it follows that

$$\frac{p(A=j)}{p(A \ge j)} = a_{0} = \frac{p(A=j+1)}{p(A \ge j+1)} $$

proving the “ ⇐” direction as well. □

## Footnotes

1 See Hartmann and Sprenger (2010) for an introduction to Bayesian epistemology with applications to topics in philosophy of science, such as explicating degree of confirmation.

2 Note that empirical adequacy does not guarantee predictive success: e.g., if *T* is a low-level theory with many parameters to be estimated simultaneously, *T* may make less accurate predictions than an empirically inadequate, but simpler theory (Forster and Sober 1994).

3 It is assumed that there are only finitely many alternative theories that satisfy these quite restrictive specifications. However, the number of alternatives may be arbitrarily large. See Dawid et al. (2015) for more discussion of that assumption.

4 The formulation *p*(*S*|*H*)=1−*𝜖* is more cautious, but this not change the qualitative results and only complicates the calculations.
